# Improvement of the Quality and the Shelf Life of the High Oxygen Modified Atmosphere Packaged Veal by Superficial Spraying with Dihydroquercetin Solution

**DOI:** 10.1155/2014/629062

**Published:** 2014-09-10

**Authors:** Stefan Georgiev Dragoev, Alexandar Stoyanov Staykov, Kiril Petrov Vassilev, Dessislav Kostadinov Balev, Dessislava Borislavova Vlahova-Vangelova

**Affiliations:** ^1^Department of Meat and Fish Technology, Technological Faculty, University of Food Technologies, 4002 Plovdiv, Bulgaria; ^2^Bulgarian Agency of Food Safety, 4400 Pazardjik, Bulgaria

## Abstract

The improvement of quality and the shelf life of veal by combination of 80%O_2_/20%CO_2_ modified atmosphere packaging and superficial spraying with 0.02% dihydroquercetin solutions was studied. The control samples C, air packaged only, D, air packaged sprayed by 0.02% dihydroquercetin solution, MAP, modified atmosphere packaging only, BMAP, modified atmosphere packaging sprayed by 0.02% butylated hydroxytoluene solution, and DMAP, modified atmosphere packaging sprayed by 0.02% dihydroquercetin solution, were measured. The best results were obtained in modified atmosphere packaging sprayed by 0.02% dihydroquercetin solution. Comparisons with control samples were expressed as reduction in acid value with 27.72%, peroxide value with 64.74%, 2-thiobarbituric acid reactive substances (TBARS) with 65.71%, and the pH with 6.18%. The acid and peroxide values, TBARS, and pH were decreased linearly in response when applying the combination of 80%O_2_/20%CO_2_ modified atmosphere packaging and superficial spraying with 0.02% dihydroquercetin solutions (*P* < 0.05). The changes of amino nitrogen content of modified atmosphere packaging veal were not influenced statistically significantly by 0.02% dihydroquercetin solution (*P* > 0.05). According to results obtained it was concluded that 80%O_2_/20%CO_2_ modified atmosphere packaged veal stored at 0 ± 0.5°C after 0.02% dihydroquercetin solution treatment can preserve its quality and shelf life to 15 d *postmortem*.

## 1. Introduction 

The fresh veal is very popular on the market. During the chilled storage, the bovine meat quality deteriorates [1]. The meat spoilage is provoked by changes in protein and lipid fractions, caused by the autolytic processes [2], growth of putrefactive microflora [3], lipid [4], and pigment oxidation [5]. High oxygen modified atmosphere packaging system induces lipid and myoglobin oxidation and protein polymerization has been shown [6]. Additionally, the relationship between ageing of beef in high oxygen modified atmosphere, calpain activity, desmin degradation, and protein oxidation has been proven, too [2]. On the one hand, lipid oxidation is a problem for the veal shelf life because it provokes haemoglobin oxidation [7]. Typically, the consumers prefer veal that is brightly red. Oxymyoglobin formed during storage on the modified atmosphere packaged veal surface gives the meat precisely brightly red colour [3]. Oxymyoglobin is not stable and during storage slowly transforms to metmyoglobin giving meat the brown grey colour [8]. On the other part, veal neck is a comparatively high fat content meat, because between muscles are arranged fatty tissue layers that transmit the marbled appearance of meat [9]. That is why the lipid oxidation is a limiting factor for shelf life of high oxygen modified atmosphere packaged veal. In this case, the main quality mechanisms that limit meat shelf life are not microbial growth but haemoglobin oxidation induced by lipid oxidation [10]. Therefore, it is crucial to preserve the quality and prolong the shelf life of high oxygen modified atmosphere packaged fresh veal [11].

The natural antioxidants have been used to increase the shelf life of different types of meat and meat products [12–14]. Natural antioxidants improve fresh meat colour [15], cooked meat flavour [11], pH, and water holding capacity [16, 17]. Many authors discussed the effect of natural antioxidants on lipid oxidation [18–22].

Many antioxidants like pentasodium tripolyphosphate [23], phytic acid [24], potassium and sodium lactate [25], sodium erythorbate, erythorbic acid, sodium L-ascorbate, L-ascorbic acid, and ascorbyl palmitate [26] were used to improve the quality of fresh beef. It was found that high concentrations of butylated hydroxytoluene inhibit lipid peroxidation [27], but it was easily absorbed in the body tissues [28]. It is effective antioxidant for minced meat [29].

An application of dihydroquercetin, extracted from Siberian larch (*Larix sibirica *Ledeb.), as an antioxidant in the surface treatment of fresh meat [4, 15, 30] and fish [31–33] has been studied too.

First Kurth and Chan [34] found the dihydroquercetin to be an effective antioxidant for lard, cottonseed oil, and butter oil. Dihydroquercetin (taxifolin) is a potent flavonoid, a member of the flavonols group [35]. By Jovanovic et al. [36] flavonoids which have a 2,4-dihydroxyacetophenone-like A ring or 2-methoxyphenol-like B ring are best electron donor and can act as chain-breaking antioxidants. The A ring can still scavenge alkyl peroxyl radicals and the superoxide radical [36]. Crawford et al. [37] suggested that the –CO–C=C– group in the pyrone ring or in the open chalcone is responsible for the antioxidant ability of flavonoids. Studying the chemical structure of dihydroquercetin these authors [37] highlighted four reasons for its antioxidant activity: (1) the double bond between C_2_ and C_3_ in combination with the keto group of the α-*β*-unsaturated ketone structure in the pyrone ring or in the corresponding chalcones is decidedly responsible for the antioxidant effect of the flavone derivatives, (2) the free (uncombined) hydroxyl group on C_3_ in the chromone ring is of decisive importance, (3) the antioxidant effect of the chromone ring system is decreased by meta hydroxyls groups, and (4) the ortho hydroxyl group on the 2-phenyl ring increases the antioxidant effect of the flavones considerably.

The antioxidant mechanism of the dihydroquercetin was described by Chen et al. [38] studying the structure-activity relationship of natural flavonoids in hydroxyl radical-scavenging effects. They [38] found the following: (1) phenolic hydroxyls in flavonoids were the main active groups capable of scavenging ^•^OH; (2) hydroxyl groups in rings B and A were important ^•^OH-scavenging active groups; (3) the ortho dihydroxyl groups in ring A and/or B could greatly enhance the ^•^OH-scavenging activity of the rings; (4) the hydroxyl groups on 3′,4′ position of ring B possessed high ^•^OH-scavenging activity and the scavenging activity of hydroxyl groups in ring B was higher than that of hydroxyl groups in ring A. (5) The structural types of flavonoids themselves could influence their ^•^OH-scavenging activity.

The effect of dihydroquercetin on peroxidation process of liposome membranes from egg phospholipids induced by ferrous sulphate or Fe^(2+)^-ascorbate system was studied too [39]. Those authors [39] suggested that the mechanism of dihydroquercetin antioxidant action consists in scavenging of lipids radicals, and its antioxidant activity matches those of α-tocopherol [39]. The effect of dihydroquercetin on these three radical-producing reactions is demonstrated [40]. It is as follows: (1) formation of superoxide by the respiratory chain, (2) formation of radicals by cytochrome c-cardiolipin complex in the presence of hydrogen peroxide or lipids, and (3) chain lipid peroxidation resulting in cytochrome c release from mitochondria and initiation of the apoptotic cascade.

Data of a wide spectrum of biological activity of dihydroquercetin are systematized [41]. Two directions of dihydroquercetin application in food industry were shown: as an antioxidant and as a biologically active supplement for creation of different types of parapharmaceutical products. Tjukavkina et al. [41] applied dihydroquercetin as efficient antioxidant with regard to vegetable oils, animal fat, milk powder, and fat containing pastry. Parapharmaceutical production with dihydroquercetin is intended for prophylactic of “oxidative stress” diseases (cardiovascular, bronchopulmonary, etc.). New benefits of dihydroquercetin application to humans are discussed in the last few years [35]. The dihydroquercetin has very low cytotoxicity [40]. That is why it has a therapeutic effect on the cancer and cardiovascular and hepatic diseases. For an explanation of its properties few mechanisms of action, including activation of the antioxidant response element and detoxifying enzymes phase II, the inhibition of cytochrome P 450 and fatty acid synthase in carcinogenesis has been discussed [35]. Kolhir et al. [42] demonstrated that dihydroquercetin not only is an antioxidant, but also possesses the properties of protecting capillaries, which is an anti-inflammatory agent and gastro- and hepatoprotecting agent, and has diuretic and hypolipidaemic activities. In addition, dihydroquercetin shows a dose dependent suppression of lipid peroxidation [42]. In this context it has been shown that the (+)-dihydroquercetin concentration dependently inhibited oxidative neuronal injuries (inhibited H_2_O_2_- and X/XO-induced neuronal injuries) and lipid peroxidation and scavenged 1,1-diphenyl-2-picrylhydrazyl free radicals [43]. It possesses antioxidant capacities, on hemolysis and platelet aggregation in human blood [44], and can reduce phospholipase C-induced hemolysis and inhibit superoxide produced by xanthine oxidase [44]. Chen and Deuster [44] suggested that the antihemolytic effects of flavonoids may not be directly mediated by removal of free radicals and may likely be due to their interaction with cell membrane.

The review of available literature sources allow us for the objective of this study to put the improving of the quality and shelf life of fresh high oxygen (80%O_2_/20%CO_2_) modified atmosphere packaged veal applying a superficial treatment with 0.02% dihydroquercetin solution.

## 2. Materials and Methods

### 2.1. Materials

The veal was supplied by Unitemp Ltd. (Voyvodinovo, Plovdiv, Bulgaria). The carcase quarters were imported from Danish Crown GB (Randers SO, Denmark) and were boned and sorted before use. The modified atmosphere packaged samples were packed in multilayer coextruded gas- and water-vapour impermeable foil with a thickness of 185 *μ*, delivered by Intrama Services GmbH, Bremen, Germany. A packaging machine Yang SR1, model Polaris VAC Ductto (Yang, Como, Italy), was used.

Powder concentrate of Siberian larch (*Larix sibirica *Ledeb.) dihydroquercetin (2R, 3R-dihydroquercetin) extract produced by Flavit Ltd. (Pushchino, Russia) was used. The concentrate contained the following: 96% dihydroquercetin, 3% dihydrokaempferol, and 1% naringenin. Two g dihydroquercetin was diluted in 25 cm^3^ 96% ethyl alcohol and filled up to 1 dm^3^ with 975 cm^3^ double distillate water.

The butylated hydroxytoluene was purchased from Merck KGaA (Darmstadt, Germany). Two g of butylated hydroxytoluene was dissolved in 100 cm^3^ 96% ethyl alcohol and filled up to 1 dm^3^ with 975 cm^3^ double distillate water.

### 2.2. Sample Preparation

The experiments were carried out with five samples, as follows: control samples (samples C)—only air packaged fresh meat; samples D—air packaged meat treated with 0.02% dihydroquercetin solution; samples MAP—modified atmosphere (80%O_2_/20%CO_2_) packaged veal only; samples BMAP—modified atmosphere (80%O_2_/20%CO_2_) packaged veal treated with 0.02% butylated hydroxytoluene solution, and samples DMAP—modified atmosphere (80%O_2_/20%CO_2_) packaged veal treated with 0.02% dihydroquercetin solution. The meat temperature during the superficial treatment was 3.2°C. Samples were strained off for 60 min at 1.2°C and after that were packaged in transparent polymer bags with size 10/28 cm. The temperature of air in the premises for packaging was 7.5°C. The packaged samples were put into plastic boxes, labelled, and stored at 0 ± 0.5°C before analysis. All samples were stored 8 days at 0 ± 0.5°C. The analyses were carried out on 7 d* post mortem* (1 d of the experiment); 11 d* post mortem* (after 4 d of storage), and 15 d* post mortem* (after 8 d of storage).

### 2.3. Methods

*Amino nitrogen content* was determined by the Sørensen formol titration method [45] based on the titration of an amino acid with formaldehyde in the presence of potassium hydroxide in the meat extract samples:

(1) RCH(NH2)COOH+HCHO+KOH  ⟶RCH(NHCH2OH)COOK+H2O

The formaldehyde reagent was prepared by diluting 25 mL of the commercial solution with 50% ethanol to a final volume of 250 mL. The pH was adjusted to 7.0 with 0.2 mol L^−1^ NaOH solution just prior to use. The pH of the veal extract was also adjusted to 7.0 with 0.2 mol L^−1^ NaOH solution. Three mL of the formaldehyde reagent was added to 3.0 mL of the veal extract and the mixture was stirred and titrated with a 0.2 mol L^−1^ NaOH solution with phenolphthalein as an indicator of its final endpoint. An excess of the 0.2 mol L^−1^ NaOH solution was added and the solution was then back-titrated with a 0.2 mol L^−1^ HCl solution until it became colorless. The required volumes of NaOH and HCl solutions were recorded.

Acid value of the extracted lipids was determined according EVS-EN ISO 660:2009 procedure [46].

The extracted lipids were dissolved in ethyl alcohol (99%) and heated for about 2 min before titrated while still hot against 0.1 M NaOH using phenolphthalein as indicator. The acid value was then calculated as follows:

(2) $$\begin{array}{c} \text{AV}=\left(56.11\times V\times N\right)\text{:}\;M, \end{array}$$

where* V* is the volume of potassium hydroxide used, mL;* N* is the exact normality;* M* is the mass of extracted lipids sample, g.

There exists an interconnection between the percentage of free fatty acids and acid value as follows:

(3) $$\begin{array}{c} \%\;\text{free}\;\text{fatty}\;\text{acid}\left(\%\text{FFA}\right)=0.503\times \text{acid}\;\text{value}\left(\text{AV}\right). \end{array}$$

pH of the samples was determined by *рН*-meter Microsyst MS 2004 (Microsyst, Plovdiv, Bulgaria), equipped with combined *рН* electrode Sensorex Combination Recorder S 450 CD (Sensorex pH Electrode Station, Garden Grove, CA, USA) [47]. The pH values of samples were reliably known to an accuracy of ±0.005. The apparatus was calibrated with standard buffer solutions (first one—potassium hydrogen phthalate standard TS with pH = 4.015 and the second one—phosphate standard buffer, TS with pH = 6.865) to check the linearity of the response of the electrode at different pH values and to detect a faulty combined electrode. The fresh standard solutions were prepared. The meat samples were cut into small pieces and weighted approximately 10 g into a blender cup. The distilled deionized water was added to volume of 100 mL. The samples were blended for 30 s on high speed and were transferred to a beaker. The pH values were read as soon as possible. Blender cups, beakers, and stir bars were rinsed in distilled water between samples. The pH electrode was rinsed with distilled water between each sample and periodically rinsed with acetone from a squeeze bottle to remove fat buildup.

Total meat lipids were extracted according to Bligh and Dyer [48] method. A hundred g of sample containing (or adjusted to contain) 75 g water (as determined by oven drying separate aliquots) was homogenized with 100 mL chloroform and 200 mL methanol (monophasic system). The solution was rehomogenized with 100 mL chloroform, following which 100 mL of either distilled water or weak salt solution (0.88% NaCl) was added. After filtration was performed under suction, the final biphasic system was allowed to separate into two layers and the lower (chloroform) phase was collected. For quantitative lipid extraction, the tissue residue was then rehomogenized with 100 mL chloroform and filtered, and the filtrate was added to the lower phase collected. Lipid content was then determined gravimetrically after evaporating a measured aliquot of the combined chloroform phase to dryness under nitrogen. The above volumes were scaled down, as long as the critical ratios of chloroform, methanol, and water (1 : 2 : 0.8 and 2 : 2 : 1.8, before and after dilution, resp.) and of initial solvent to tissue [(3 + 1) : 1] were kept identical.

Peroxide value of the extracted lipids was determined using ISO 3960:2007 procedure described by Djenane et al. [11]. The test sample was first dissolved in mixture of chloroform and acetic acid (2 : 3). By flowing nitrogen gas through the sample the residual oxygen was dispelled. The saturated potassium iodide was added. The free iodine was titrated with 0.01 mol/L sodium thiosulfate (*f* = 1.006). The endpoint was determined by the maximum inflexion point on titration curve. Peroxide value was calculated from titration volume of sodium thiosulfate as follows:

(4) $$\begin{array}{c} {\text{I}}_{2}+2{\text{Na}}_{2}{\text{S}}_{2}{\text{O}}_{3}⟶{\text{Na}}_{2}{\text{S}}_{4}{\text{O}}_{6}+2\text{NaI} \end{array}$$

Approximately 5 g sample was delivered into a conical flask with stopper. 30 mL solvent was added and gently shaken to dissolve the sample completely. The air inside flask was gently replaced with nitrogen to remove remaining oxygen. By further flowing nitrogen gas, the 0.5 mL saturated potassium iodide was added, and the flask was immediately sealed and gently was shaken for 1 min. The flask was left at room temperature 15–20°C in a dark room. The 30 mL pure water was added and was sealed and stirred. The peroxide value was measured by titration with 0.01 mol/L sodium thiosulfate. Likewise, blank level was obtained in advance by a blank test. Peroxide value (meqv O_2_ kg^−1^) was calculated using

(5) $$\begin{array}{c} \text{POV}=\left({\mathrm{EP}}_{1}-{\text{BL}}_{1}\right)\times \text{TF}\times \frac{R}{W}, \end{array}$$

where EP_1_ is titration volume, mL; BL_1_ is blank level (0.00 mL); TF is factor of reagent (1.006);* R* is constant (10); and* W* is sample weight, g.

TBARS was determined by the method of Botsoglou et al. [49] using double beam UV-vis spectrophotometer Camspec, model M 550 (Camspec Ltd., Cambridge, United Kingdom). A 2 g sample was transferred into a 25 mL centrifuge tube, and volumes of 5% aqueous TCA (8 mL) and 0.8% BHT in hexane (5 mL) were successively added. The content of the tube was ultraturraxed for 30 s at high speed and centrifuged for 3 min at 3000 g, and the top hexane layer was discarded. The bottom aqueous layer was made to 10 mL volume with 5% TCA, and a 2.5 mL aliquot was pipetted into a screw-capped tube to which a volume (1.5 mL) of 0.8% aqueous TBA was also added. Following incubation for 30 min. at 70°C, the tube was cooled under tap water, and the reaction mixture was submitted to third derivative spectrophotometry against blank reaction mixture.

Aliquots of standard solution were pipetted into screw-capped tubes and diluted to 2.5 mL volume with 5% TCA. A 1.5 mL volume of 0.8% TBA was added in each tube, and the reaction was carried out as prescribed. Calibration curves were constructed by plotting values of peak height at 521.5 nm, as they are printed in the instrumental chart in arbitrary units, versus known concentration of MDA in the final reaction mixture. The concentration of MDA in the sample extracts was calculated on the basis of the slope and intercept data of the computed least-squares calibration curves. In case the absorbance value exceeded 1.0, sample extract was appropriately diluted with water before final measurement. MDA was determined in samples using

(6) $$\begin{array}{c} \text{MDA}\;\text{content},\text{ppb}=16\;C\times V\text{:}\;W, \end{array}$$

where* C* is a MDA concentration (ng/mL) in the sample extract according to the calibration curve,* V* is dilution factor of sample extract, and* W* is the weight (g) of the sample.

For identification of fatty acid compositions of the total lipids as fatty acid methyl esters all veal samples were analyzed after eight days of storage at 0 ± 0.5°C. For this purpose a gas-chromatograph Agilent 6890 Plus (Agilent Technologies, Santa Clara, CA, USA) was used. It was equipped with 5793 mass-selective detector (Agilent Technologies, Santa Clara, CA, USA) and 30 m × 0.25 mm × 0.25 *µ*m SP 2380 capillary column (Supelco, Bellefonte PA, USA) at the following conditions: gradient of the temperature from 150°*С* to 230°*С* with speed 3°*С*/min and 15 min standing at final temperature; the injector temperature −260°*С*; the detector temperature −280°*С*; carrier gas, helium with flow speed 0.8 cm^3^/min; the injected volume 1.5 *µ*L; and split −20 : 1. The mass-spectral detector was operated at the temperatures: *T*_quad_ = 150°C and *T*_source_ = 230°C. The fatty acid residues were identified compared to trade standards [50]. The results were presented in relative percentages area of the relevant peaks in the chromatograms, as obtained from the integrator.

The microbiological analyses were performed as follows:* Listeria monocytogenes* according to ISO 11290-2002 [51];* Salmonella *spp. according to ISO 6579-2002 [52];* Escherichia coli* according to ISO 16649-1:2001 [53]; total aerobic colony count according to EN ISO/DIS 4833-2001 [54].

Data was statistically analyzed by SPSS 11.0 software (SPSS Inc., Chicago, Illinois, USA). Nine repetitions (*n* = 9) for each sample were carried out. Data were processed by the analysis of variance method with a level of significance of *P* < 0.05 [55]. Duncan's multiple comparison test (SPSS) with a significant difference set at *P* ≥ 0.05 was used to compare sample means. Significant differences between means less than 0.05 were considered statistically significant [56].

## 3. Results and Discussion

### 3.1. Amino Nitrogen Content

At all examined samples gradual and statistically significant (*P* < 0.05) increase of the amino nitrogen content during 8 days of storage at 0 ± 0.5°C was found (Table 1). On the end of the experiments the amino nitrogen content of the control samples C increased 6.8 times, of samples D 6.6 times, of samples MAP 6.4 times, and of samples BMAP and DMAP 6.3 times respectively. The comparisons between amino nitrogen content of the five examined samples on 1, on 4, and on 8 days of storage, respectively, were done. No statistically significant differences (*P* > 0.05) of amino nitrogen content were evaluated. After 8 days of storage the amino nitrogen content of every one of veal samples does not exceed the limit of 10 mg/100 g meat. The obtained results showed that expected proteolysis [57] of refrigerated veal storage to 15 d* post mortem* at 0 ± 0.5°C existed. The proteolysis of meat was known and expected phenomenon [2]. The similarity of our results was reported by Feidt et al. [58] which found the increases of free amino acid amounts in meat stored at 4°C to 14 d* post mortem*. It was concluded that the factors modified atmosphere packaging and antioxidant treatments did not influence the veal proteolysis.

### 3.2. Acid Value

The clearly pronounced lipolysis of total veal lipids in all examined samples was found (Table 1). In all examined samples a statistically significant (*P* < 0.05) increase of acid value (i.e., increase of free fatty acid content) during 8 days of storage was determined. The increases in different samples was as follows: in samples DMAP—4.75 times, in samples BMAP—4.89 times, in control samples C—6.57 times, in samples MAP—6.60 times, and in samples D—7.16 times, respectively (Table 1). Those results indicated that the veal lipids undergo depth lipolysis during meat refrigerated storage independently of type of packaging and superficial antioxidant treatments. At first sight results obtained are strange. Although in our previous works [30] we expressed similar changes of acid value of modified atmosphere packaged beef trimmings 90/10% and beef knuckle with bones sprayed with dihydroquercetin solution on 18th day of storage at 0–4°C, Karpińska-Tymoszczyk [13, 21] also found a similar phenomenon and established lower hydrolytic changes in turkey meatballs stored at 0–4°C when the sodium erythorbate was added [13, 21]. A comparison of the indicators pH, peroxide value, and TBARS in Table 1 shows similar trends of changes of those determined about acid value. Therefore, the realization is dawning that there is some correlation between hydrolytic and oxidative identified changes in the lipid fraction of packaged under modified atmosphere and treated with antioxidants veal. Explanation of the results may be sought in the composition and properties of the natural extract of Siberian larch. On the one hand, it is not a pure substance [36, 39], on the assumption that the polyphenol structures dihydroquercetin and butylated hydroxytoluene probably exhibit some degree of inhibitory effect both on the muscle [11] and on the microbial lipase systems [1]. However, this hypothesis is very brave and future studies are needed to be carried out to confirm or reject it.

### 3.3. pH Analysis

Comparatively low pH was found at all samples such as on 1 day of storage and on 8 days of storage (Table 1). At the end of the experiment pH of all examined samples was increased slightly but statistically significantly (*P* < 0.05) (Table 1). The increases of the pH of samples treated with 0.02% antioxidants were lower than in the other samples. (BMAP by only 2.99% and DMAP up with 4.86%), while in control samples C pH was grown up with 18.79% (Table 1). Those results give us a reason to assume that application of antioxidant solutions probably supported growth of the lactic acid bacteria and thus contributed to maintaining of lower meat pH. The role of lactic acid spoilage bacteria such as* Pseudomonas* spp. and* Lactobacillus sakei* in beef stored at 5°C under 60%O_2_/40%CO_2_ modified atmosphere packaging conditions [1] and* Photobacterium* spp. occurring in beef stored at 4°C in air [59] was reported earlier. Another reason for the relatively lower pH values found on 1 day of storage (Table 1) perhaps is a use of pinkish red soft and exudative meat in the experiment [8]. This hypothesis was based on the detected pale purple red colour, soft texture, and very exudative veal, which are more typical for meat in stage of rigor mortis than in the initial autolysis.

### 3.4. Peroxide Value

The peroxide value of all examined samples increasing statistically significantly (*P* < 0.05) after 8 days of storage at 0 ± 0.5°C was determined (Table 1). These data are evidences for lipid hydroperoxides (the primary lipid peroxidation products) formation in veal meat. During 8 days of storage a significant increase of peroxide value of samples MAP, C, and D was found. Peroxide value of control sample C increased by 70.18% (Table 1). In the same time the peroxide value of samples D increased by 67.92% and of samples MAP by 69.08%. The peroxide value of samples MAP, C, and D on the end of storage were not statistically significantly different (*P* > 0.05). After 8 days of storage at 0 ± 0.5°C, almost samples BMAP increased by 48.38%, but samples DMAP just with 15.46% (Table 1). Obviously, the antioxidants treatment of modified atmosphere packaged veal, and in particular dihydroquercetin, contributes to a significant reduction of peroxide value. The similar results were reported by Gurinovich et al. [29] which were found that dihydroquercetin significantly inhibited the oxidation process of minced meat. The growth of the primary oxidation products can be explained with chelation capacity of dihydroquercetin [37] and it's hydroxyl radical-scavenging effects [38]. This is the reason for inhibition action of dihydroquercetin against free radical formation [39] during the early stages of storage. The amount of peroxides in the lipid fraction rest at an acceptable level and even prolongs the shelf life of meat [29]. In addition, the effective stabilization of lipids against oxidation was determined by Vladimirov et al. [40].

### 3.5. TBARS

The TBARS growth of all studied samples during their 8-day storage was found (Table 1). In the end of experiment TBARS increased as follows: at control samples C—4.29 times; at samples D—1.88 times, at samples MAP—1.41 times, at samples BMAP—1.32 times; and at samples DMAP—1.47 times (*P* < 0.05). After 8 days of storage the TBARS of samples MAP, DMAP, and BMAP were not significantly (*P* ≥ 0.05) different (Table 1). Excluding the control samples C, it was estimated that the TBARS of four experimental samples vary in range 0.35–0.64 mg malondialdehyde/kg meat, which is lower than the limit of 1.00 mg malondialdehyde/kg meat discussed as a critical limit for fresh meat [60]. Our results are similar to the results of Gatellier et al. [19] investigating effect of α-tocopherol acetate supplementation on 80%O_2_/20%CO_2_ modified atmosphere packaged beef stored under refrigerated 13 d at 8°C, of Djenane et al. [11] examining the antioxidant mixture of rosemary and vitamin C together with 70%O_2_/20%CO_2_/10%N_2_ modified atmosphere packaging of fresh beef steaks, and of Bakalivanova and Kaloyanov [18] determining a statistically significant TBARS reduction of mechanically separated poultry meat when 120 mg/kg taxifolin (dihydroquercetin) and 400 mg/kg rosemary oleoresin extract were applied. As typical scavengers of hydroxyl radicals, dihydroquercetin and butylated hydroxytoluene inhibit free radical formation [38–40] and act as suitable antioxidants against development and distribution of lipid oxidation secondary products [37, 41].

### 3.6. Fatty Acid Composition

The fatty acid compositions of the lipids extracted from veal samples did not significantly (*P* ≥ 0.05) change during refrigerated storage. (Table 2).

### 3.7. Microbiological Status

The results of microbiological analysis of veal neck in dynamics showed that all samples meet the requirements of Commission Regulation (EC) no. 1441/2007 [61] (Table 3). It was found that total aerobic count of mesophilic microorganisms was between 5 · 10^5^ cfu/g and 5 · 10^6^ cfu/g,* Escherichia coli* was between 500 cfu/g and 5000 cfu/g, and* Salmonella spp.* was not detected in 25 g of the meat. No presence of* Listeria monocytogenes* in 1 g samples in 15 d* post mortem* veal stored at 0 ± 0.5°C was found. Samples DMAP preserved their microbial quality after 8 days of storage at 0 ± 0.5°C. For comparison the control samples C were saved to consume up to 1 day of storage and the sample D to 4 days of storage. The results obtained allow us to conclude that 80%O_2_/20%CO_2_ modified atmosphere packaging was crucial for extension of the shelf life in maintaining a relatively constant temperature 0 ± 0.5°C. Surface treatment with 0.02% dihydroquercetin did not affect microbial growth and the shelf life of fresh veal. According to the results obtained by microbiological test, veal neck wrapped in 80%O_2_/20%CO_2_ modified atmosphere package can be stored 4 d more than air packaged veal. Our data find good explanations by the results for microbial spoilage of antioxidant treated and high oxygen modified atmosphere or air packaged beef, stored at 0–5°C [1, 11, 21, 53, 59].

## 4. Conclusion

The combination of 80%O_2_/20%CO_2_ modified atmosphere packaging and superficial spraying with 0.02% dihydroquercetin solution can be used to improve veal quality and to extend the shelf life to reduce the acid value with 27.72%, the pH with 6.18%, the peroxide value with 64.74%, and the TBARS with 65.71% and to save the microbiological status of meat to 15 d* post mortem* at 0 ± 0.5°C.

## Figures and Tables

**Table 1:** The changes of veal quality parameters (amino nitrogen content, acid value, pH, peroxide value, and TBARS) during 8 days of storage at 0 ± 0.5°C.

	Samples C			Samples D			Samples MAP			Samples ВMAP			Samples DMAP		
	1 d	4 d	8 d	1 d	4 d	8 d	1 d	4 d	8 d	1 d	4 d	8 d	1 d	4 d	8 d
Amino nitrogen content, mg/100 g meat	0.73^a^ ± 0.14	3.85^b^ ± 0.15	4.95^c^ ± 0.20	0.73^a^ ± 0.14	3.77^b^ ± 0.16	4.81^c^ ± 0.19	0.73^a^ ± 0.14	3.65^b^ ± 0.18	4.67^c^ ± 0.13	0.73^a^ ± 0.14	3.54^b^ ± 0.19	4.59^c^ ± 0.16	0.73^a^ ± 0.14	3.56^b^ ± 0.20	4.61^c^ ± 0.15
Acid value, mg KOH/100 g lipids	0.28^a^ ± 0.09	1.39^c^ ± 0.11	1.84^e^ ± 0.14	0.28^a^ ± 0.09	1.50^c^ ± 0.17	2.13^f ^± 0.15	0.28^a^ ± 0.09	1.17^c^ ± 0.16	1.85^e^ ± 0.14	0.28^a^ ± 0.09	0.83^b^ ± 0.13	1.37^d^ ± 0.11	0.28^a^ ± 0.09	0.75^b^ ± 0.10	1.33^d^ ± 0.10
pH value	5.35^a^ ± 0.04	5.56^c^ ± 0.04	5.98^e^ ± 0.05	5.35^a^ ± 0.04	5.49^b^ ± 0.03	5.62^c^ ± 0.03	5.35^a^ ± 0.04	5.39^b^ ± 0.05	5.75^d^ ± 0.03	5.35^a^ ± 0.04	5.36^a^ ± 0.02	5.51^b^ ± 0.05	5.35^a^ ± 0.04	5.52^b^ ± 0.03	5.61^c^ ± 0.03
Peroxide value, meqv O_2_/kg lipids	0.35^a^ ± 0.07	0.75^c^ ± 0.06	1.17^d^ ± 0.11	0.35^a^ ± 0.07	0.66^c^ ± 0.06	1.09^d^ ± 0.10	0.35^a^ ± 0.07	0.83^c^ ± 0.09	1.13^d^ ± 0.09	0.35^a^ ± 0.07	0.44^b^ ± 0.08	0.68^c^ ± 0.09	0.35^a^ ± 0.07	0.38^b^ ± 0.05	0.41^b^ ± 0.04
TBARS, mg MDA/kg meat	0.36^a^ ± 0.06	0.50^b^ ± 0.04	1.53^d^ ± 0.04	0.36^a^ ± 0.06	0.37^a^ ± 0.05	0.67^c^ ± 0.08	0.36^a^ ± 0.06	0.41^a^ ± 0.05	0.51^b^ ± 0.04	0.36^a^ ± 0.06	0.38^a^ ± 0.07	0.47^a^ ± 0.05	0.36^a^ ± 0.06	0.40^a^ ± 0.04	0.53^b^ ± 0.04

Means ± standard deviation. Different letters (a, b, c, d, e, and f) in the rows indicate significant differences for each parameter (*P* < 0.05). Sample denomination: control samples (samples C)—only air packaged fresh veal; the experimental samples: samples D—air packaged veal treated with 0.02% dihydroquercetin solution; samples MAP—modified atmosphere (80%O_2_/20%CO_2_) packaged veal only; samples BMAP—modified atmosphere (80%O_2_/20%CO_2_) packaged veal treated with 0.02% butylated hydroxytoluene solution; and samples DMAP—modified atmosphere (80%O_2_/20%CO_2_) packaged veal treated with 0.02% dihydroquercetin solution.

**Table 2:** The changes of FAME composition of veal samples during 8 DOS (15 d *post mortem*) at 0 ± 0.5°C.

Fatty acid methyl esters	Samples C	Samples D	Samples MAP	Samples BMAP	Samples DMAP
Myristic acid C14:0	2.72 ± 0.35	2.41 ± 0.33	2.40 ± 0.37	2.22 ± 0.31	2.28 ± 0.28
Pentadecanoic acid C15:0	0.43 ± 0.11	0.51 ± 0.12	0.42 ± 0.10	0.48 ± 0.09	0.43 ± 0.11
Palmitic acid C16:0	25.98 ± 0.49	23.96 ± 0.46	23.67 ± 0.50	25.63 ± 0.52	22.85 ± 0.42
Heptadecanoic acid C17:0	2.26 ± 0.32	2.46 ± 0.29	2.31 ± 0.30	2.16 ± 0.28	2.22 ± 0.31
Stearic acid C18:0	14.33 ± 0.35	14.29 ± 0.33	14.82 ± 0.37	13.79 ± 0.32	13.60 ± 0.39
Nonadecanoic acid C19:0	Traces	Traces	Traces	Traces	Traces
Behenic acid C20:0	0.64 ± 0.22	0.69 ± 0.27	0.50 ± 0.29	1.37 ± 0.24	1.77 ± 0.28
Miristoleinic acid C14:1 cis-9	0.52 ± 0.16	0.45 ± 0.15	0.44 ± 0.17	0.62 ± 0.19	0.47 ± 0.13
Palmitoleic acid C16:1 cis-9	4.17 ± 0.29	3.51 ± 0.33	3.78 ± 0.36	4.62 ± 0.35	3.68 ± 0.32
Heptadecenoic acid C17:1 cis-10	0.93 ± 0.27	1.25 ± 0.24	1.09 ± 0.29	0.97 ± 0.28	1.12 ± 0.21
Oleic acid C18:1 cis-9	33.60 ± 0.38	38.05 ± 0.37	36.47 ± 0.40	35.77 ± 0.34	34.37 ± 0.39
Elaidinic acid C18:1 trans-9	3.14 ± 0.19	0.08 ± 0.02	3.12 ± 0.15	1.87 ± 0.32	3.73 ± 0.22
Vaccenic acid C18:1 cis-11	1.97 ± 0.17	2.72 ± 0.11	2.17 ± 0.17	1.89 ± 0.21	1.79 ± 0.19
Trans-vaccenic acid C18:1 trans-11	4.32 ± 0.31	3.97 ± 0.25	3.13 ± 0.21	2.81 ± 0.36	3.71 ± 0.26
Erucic acid C22:1 cis-13	Traces	Traces	Traces	Traces	Traces
Linoleic acid *ω*-6 9, 12-C18:2	3.29 ± 0.28	3.80 ± 0.24	4.15 ± 0.33	4.20 ± 0.32	3.82 ± 0.26
Eicosadienoic acid *ω*-6 9, 12-C20:2	0.31 ± 0.10	0.32 ± 0.10	0.10 ± 0.04	0.10 ± 0.03	0.60 ± 0.17
Eicosatetraenoic acid *ω*-6 8-, 11-, 14-C20:3	0.35 ± 0.13	0.31 ± 0.15	0.10 ± 0.04	0.10 ± 0.03	0.82 ± 0.20
Arachidonic acid *ω*-6 8-, 11-, 14-, 17-C20:4	0.44 ± 0.16	0.74 ± 0.13	0.79 ± 0.19	0.66 ± 0.18	1.83 ± 0.17
Conjugated linoleic acid (CLA) cis-9, trans-11 C18: 2	Traces	Traces	Traces	Traces	Traces
Linolenic acid *ω*-3 9-, 12-, 15-C 18:3	0.56 ± 0.14	0.41 ± 0.11	0.50 ± 0.15	0.72 ± 0.18	0.85 ± 0.16

Total	99.96 ± 0.18	99.93 ± 0.20	99.96 ± 0.17	99.98 ± 0.19	99.94 ± 0.21

SFA	46.36%	44.32%	44.12%	45.65%	43.15%
MUFA	48.65%	50.03%	50.20%	48.55%	48.87%
PUFA	4.95%	5.58%	5.64%	5.78%	7.92%
*ω*-6/*ω*-3 PUFA	0.12	0.08	0.10	0.14	0.12
PUFA/SFA	0.107	0.126	0.128	0.127	0.184

**Table 3:** Microbiological status of veal samples during 8 DOS (15 d *post mortem*) at 0 ± 0.5°C.

Samples	Shelf life, DOS	Total mesophilic aerobic and facultative anaerobic microorganisms log cfu/g	*Escherichia coli*, log cfu/g	*Salmonella *spp., absence in 25 g	*Listeria monocytogenes*, absence in 1 g
	Norms	5.10^5^–5.10^6^ cfu/g or 5.7–6.7 log cfu/g	500–5000 cfu/g or 2.7–3.7 log cfu/g	Not to be detected in 25 g of meat	Not to be detected in 1 g of meat
Samples C	1 d (7 d *post mortem*)	2.7 ± 1.1	1.3 ± 0.6	—	—
	4 d (11 d *post mortem*)	4.6 ± 1.7	2.1 ± 0.5	—	1
	8 d (15 d *post mortem*)	6.5 ± 1.6	2.2 ± 0.5	—	2

Samples D	1 d (7 d *post mortem*)	2.8 ± 1.6	1.4 ± 0.5	—	—
	4 d (11 d *post mortem*)	3.5 ± 1.3	1.9 ± 0.8	—	—
	8 d (15 d *post mortem*)	4.4 ± 1.7	2.1 ± 0.4	—	1

Samples MAP	1 d (7 d *post mortem*)	2.4 ± 1.0	1.5 ± 0.6	—	—
	4 d (11 d *post mortem*)	2.9 ± 1.4	1.5 ± 0.6	—	—
	8 d (15 d *post mortem*)	3.9 ± 1.8	1.7 ± 0.7	—	—

Samples ВMAP	1 d (7 d *post mortem*)	2.5 ± 1.2	1.4 ± 0.6	—	—
	4 d (11 d *post mortem*)	2.8 ± 1.1	1.5 ± 0.7	—	—
	8 d (15 d *post mortem*)	3.1 ± 1.5	1.8 ± 0.4	—	—

Samples DMAP	1 d (7 d *post mortem*)	2.6 ± 0.9	1.6 ± 0.6	—	—
	4 d (11 d *post mortem*)	2.8 ± 1.4	1.7 ± 0.6	—	—
	8 d (15 d *post mortem*)	3.3 ± 1.2	1.8 ± 0.5	—	—

## References

[1] Ercolini D., Russo F., Torrieri E., Masi P., Villani F. (2006). Changes in the spoilage-related microbiota of beef during refrigerated storage under different packaging conditions. *Applied and Environmental Microbiology*.

[2] Lindahl G., Lagerstedt Å., Ertbjerg P., Sampels S., Lundström K. (2010). Ageing of large cuts of beef loin in vacuum or high oxygen modified atmosphere—effect on shear force, calpain activity, desmin degradation and protein oxidation. *Meat Science*.

[3] Eeckhaut V., Boyen F., Pasmans F. (2012). *Clostridium novyi* type B as a causative agent of bovine meat spoilage. *Anaerobe*.

[4] Ivanov G. Y., Staykov A. S., Balev D. K. (2010). Effect of treatment with natural antioxidant on the chilled beef lipid oxidation. *Advance Journal of Food Science and Technology*.

[5] Jakobsen M., Bertelsen G. (2000). Colour stability and lipid oxidation of fresh beef. Development of a response surface model for predicting the effects of temperature, storage time, and modified atmosphere composition. *Meat Science*.

[6] Kim Y. H., Huff-Lonergan E., Sebranek J. G., Lonergan S. M. (2010). High-oxygen modified atmosphere packaging system induces lipid and myoglobin oxidation and protein polymerization. *Meat Science*.

[7] Staykov A., Balev D., Vasilev K., Dragoev S., Vlahova-Vangelova D. (2013). Stabilisation of sensory properties, colour and conjugated lipid products in modified atmosphere packaged veal, treated with dihydroquercetin. *Food Processing Industry Magazine*.

[8] Mancini R. A., Hunt M. C. (2005). Current research in meat color. *Meat Science*.

[9] Jones S. D. M., Jeremiah L. E., Tong A. K. W., Robertson W. M., Lutz S. (1991). The effects of marbling level, electrical stimulation, and postmortem aging on the cooking and palatability properties of beef rib-eye steaks. *Canadian Journal of Animal Science*.

[10] Dragoev S. (2011). Chemistry of the lipid peroxidation in meat and meat products. *Meat and Meat Products*.

[11] Djenane D., Sánchez-Escalante A., Beltrán J. A., Roncalés P. (2003). Extension of the shelf life of beef steaks packaged in a modified atmosphere by treatment with rosemary and displayed under UV-free lighting. *Meat Science*.

[12] Bakalivanova T., Grigorova S., Kaloyanov N. (2009). Effect of irradiation and packaging on lipid fraction of Bulgarian salami during storage. *Radiation Physics and Chemistry*.

[13] Karpińska-Tymoszczyk M. (2011). The effect of water-soluble rosemary extract, sodium erythorbate, their mixture and packaging method on the quality of Turkey meatballs. *Italian Journal of Food Science*.

[14] Rohlík B.-A., Pipek P. (2012). Rosemary extract and its affect on meat products' properties. *Fleschwirtschaft International*.

[15] Balev D. K., Staykov A. S., Ivanov G. Y., St. Dragoev G., Filizov E. H. (2011). Color stability improvement of chilled beef by natural antioxidant treatment and modified atmosphere packaging. *American Journal of Food Technology*.

[16] McBride N. T. M., Hogan S. A., Kerry J. P. (2007). Comparative addition of rosemary extract and additives on sensory and antioxidant properties of retail packaged beef. *International Journal of Food Science and Technology*.

[17] Rojas M. C., Brewer M. S. (2007). Effect of natural antioxidants on oxidative stability of cooked, refrigerated beef and pork. *Journal of Food Science*.

[18] Bakalivanova T., Kaloyanov N. (2012). Effect of taxifolin, rosemary and synthetic antioxidants treatment on the poultry meat lipid peroxidation. *Comptes Rendus de L'Academie Bulgare des Sciences*.

[19] Gatellier P., Hamelin C., Durand Y., Renerre M. (2001). Effect of a dietary vitamin E supplementation on colour stability and lipid oxidation of air- and modified atmosphere-packaged beef. *Meat Science*.

[20] Karpińska-Tymoszczyk M. (2010). The effect of sage, sodium erythorbate and a mixture of sage and sodium erythorbate on the quality of turkey meatballs stored under vacuum and modified atmosphere conditions. *British Poultry Science*.

[21] Karpińska-Tymoszczyk M. (2013). The effect of oil-soluble rosemary extract, sodium erythorbate, their mixture, and packaging method on the quality of Turkey meatballs. *Journal of Food Science and Technology*.

[22] Rohlik B.-O., Pipek P., Panek J. (2010). The effect of natural antioxidants on the colour of dried/cooked sausages. *Czech Journal of Food Science*.

[23] Brewer S. Preserving beef quality with natural antioxidants. http://www.beefresearch.org/CMDocs/BeefResearch/PE_White_%20Papers/Preserving_Beef_with_Natural_Antioxidants.pdf.

[24] Stodolak B., Starzyńska A., Czyszczoń M., Zyła K. (2006). The effect of phytic acid on oxidative stability of raw and cooked meat. *Food Chemistry*.

[25] Knock R. C., Seyfert M., Hunt M. C. (2006). Effects of potassium lactate, sodium chloride, sodium tripolyphosphate, and sodium acetate on colour, colour stability, and oxidative properties of injection-enhanced beef rib steaks. *Meat Science*.

[26] Sepe H. A., Faustman C., Lee S., Tang J., Suman S. P., Venkitanarayanan K. S. (2005). Effects of reducing agents on premature browning in ground beef. *Food Chemistry*.

[27] Ozer O., Sariçoban C. (2010). The effects of butylated hydroxyanisole, ascorbic acid, and α-tocopherol on some quality characteristics of mechanically deboned chicken patty during freeze storage. *Czech Journal of Food Sciences*.

[28] Babu B., Wu J.-T. (2008). Production of natural butylated hydroxytoluene as an antioxidant by freshwater phytoplankton. *Journal of Phycology*.

[29] Gurinovich G. V., Lissin K. V., Potipaeva N. N. (2005). Preparation for increasing storage life of comminuted meat product. *Meat Industry*.

[30] Ivanov G. Y., Staykov A. S., Balev D. K. (2010). Effect of natural antioxidant treatment and modified atmosphere packaging on the quality and shelf-life of chilled beef. *Agriculture and Biology Journal of North America*.

[31] Ivanov G., Balev D., Nikolov H., Dragoev S. (2009). Improvement of the chilled salmon sensory quality by pulverisation with natural dihydroquercetin solutions. *Bulgarian Journal of Agricultural Science*.

[32] Balev D., Ivanov G., Nikolov H., Dragoev S. (2009). Effect of natural antioxidant pre-treatment on the properties of colour surface of chilled-stored salmon discs. *Bulgarian Journal of Agricultural Science*.

[33] Dragoev S. G., Balev D. K., Ivanov G. Y. (2014). Effect of superficial treatment with new natural antioxidant on salmon (Salmo salar) lipid oxidation. *Acta Alimentaria*.

[34] Kurth E. F., Chan F. L. (1951). Dihydroquercetin as an antioxidant. *Journal of the American Oil Chemists' Society*.

[35] Weidmann A. E. (2012). Dihydroquercetin: more than just an impurity?. *European Journal of Pharmacology*.

[36] Jovanovic S. V., Steenken S., Hara Y., Simic M. G. (1996). Reduction potentials of flavonoid and model phenoxyl radicals. Which ring in flavonoids is responsible for antioxidant activity?. *Journal of the Chemical Society. Perkin Transactions*.

[37] Crawford D. L., Sinnhuber R. O., Aft H. (1961). The effect of methylation upon the antioxidant and chelation capacity of quercetin and dihydroquercetin in a lard substrate. *Journal of Food Science*.

[38] Chen J. W., Zhu Z. Q., Hu T. X., Zhu D. Y. (2002). Structure-activity relationship of natural flavonoids in hydroxyl radical-scavenging effects. *Acta Pharmacologica Sinica*.

[39] Teselkin I. O., Zhambalova B. A., Babenkova I. V., Tiukavkina N. A. (1996). Antioxidant properties of dihydroquercetin. *Biophysics*.

[40] Vladimirov Y. A., Proskurnina E. V., Demin E. M. (2009). Dihydroquercetin (taxifolin) and other flavonoids as inhibitors of free radical formation at key stages of apoptosis. *Biochemistry*.

[41] Tjukavkina N. A., Rulenko I. A., Kolesnik Y. A. (1997). Dihydroquercetin—a new antioxidant and biologically active food supplement. *Issues of Nutrition*.

[42] Kolhir V. K., Bykov V. A., Baginskaja A. I. (1996). Antioxidant activity of a dihydroquercetin isolated from Larix gmelinii (Rupr.) Rupr. Wood. *Phytotherapy Research*.

[43] Dok-Go H., Lee K. H., Kim H. J. (2003). Neuroprotective effects of antioxidative flavonoids, quercetin, (+)-dihydroquercetin and quercetin 3-methyl ether, isolated from *Opuntia ficus-indica* var. *saboten*. *Brain Research*.

[44] Chen Y., Deuster P. (2009). Comparison of quercetin and dihydroquercetin: antioxidant-independent actions on erythrocyte and platelet membrane. *Chemico-Biological Interactions*.

[45] Tešanović D., Kalenjuk B., Tešanović D., Psodorov Đ., Ristić Z., Marković V. (2011). Changes of biochemical and sensory characteristics in the muscles longissimus dorsi of the fallow deer in the early phase post-mortem and during maturation. *African Journal of Biotechnology*.

[46] Kardash E., Tur'yan Y. I. (2005). Acid value determination in vegetable oils by indirect titration in aqueous-alcohol media. *Croatica Chemica Acta*.

[47] Korkeala H., Mäki-Petäys O., Alanko T., Sorvettula O. (1986). Determination of pH in meat. *Meat Science*.

[48] Bligh E. G., Dyer W. J. (1959). A rapid method of total lipid extraction and purification. *Canadian Journal of Biochemistry and Physiology*.

[49] Botsoglou N. A., Fletouris D. J., Papageorgiou G. E., Vassilopoulos V. N., Mantis A. J., Trakatellis A. G. (1994). Rapid, sensitive, and specific thiobarbituric acid method for measuring lipid peroxidation in animal tissue, food, and feedstuff samples. *Journal of Agricultural and Food Chemistry*.

[50] Christie W. (1984). Extraction and hydrolysis of lipids and some reactions of their fatty acid components. *CRC Handbook of Chromatography: Lipids*.

[51] Scotter S. L., Langton S., Lombard B. (2001). Validation of ISO method 11290. Part 1—detection of Listeria monocytogenes in foods. *International Journal of Food Microbiology*.

[52] Piknová L., Štefanovicová A., Drahovská H., Sásik M., Kuchta T. (2002). Detection of *Salmonella* in food equivalent to ISO 6579, by a three-days polymerase chain reaction-based method. *Food Control*.

[53] Nastasijevic I., Mitrovic R., Buncic S. (2009). The occurrence of *Escherichia coli* O157 in/on faeces, carcasses and fresh meats from cattle. *Meat Science*.

[54] Cohen N., Ennaji H., Bouchrif B., Hassar M., Karib H. (2007). Comparative study of microbiological quality of raw poultry meat at various seasons and for different slaughtering processes in Casablanca (Morocco). *The Journal of Applied Poultry Research*.

[55] Draper N. R., Smith H. (1998). *Applied Regression Analysis*.

[56] Kenward M. G. (1987). A method for comparing profiles of repeated measurements. *Applied Statistic*.

[57] Sosulski F. W., Imafidon G. I. (1990). Amino acid composition and nitrogen-to-protein conversion factors for animal and plant foods. *Journal of Agricultural and Food Chemistry*.

[58] Feidt C., Petit A., Bruas-Reignier F., Brun-Bellut J. (1996). Release of free amino-acids during ageing in bovine meat. *Meat Science*.

[59] Pennacchia C., Ercolini D., Villani F. (2011). Spoilage-related microbiota associated with chilled beef stored in air or vacuum pack. *Food Microbiology*.

[60] Koch A. G., Christensen H., Eides S. P., Meinert L. Shelf life requirements for fresh meat and meat products.

[61] (2007). Commission Regulation (EC) No 1441/2007 of 5 December 2007 amending Regulation (EC) No 2073/2005 on microbiological criteria for foodstuffs. *Official Journal of Eurropean Union*.

